# Anti-Migration Effects of *Gekko* Sulfated Glycopeptide on Human Hepatoma SMMC-7721 Cells

**DOI:** 10.3390/molecules16064958

**Published:** 2011-06-16

**Authors:** Xiong-Zhi Wu, Dan Chen, Xiao-Qiang Han

**Affiliations:** 1 Zhong-Shan-Men In-Patient Department, Tianjin Medical University Cancer Institute and Hospital, HuaN–Hu-Xi-Road, He-Xi-District, Tianjin 300060, China; Email: ilwxz@163.com; 2 Department of Pharmacology, School of Basic Medical Sciences, Tianjin Medical University, Tianjin 300070, China; 3 State Key Laboratory of Phytochemistry and Plant Resources in West China (CUHK), Institute of Chinese Medicine, The Chinese University of Hong Kong, Shatin, N. T., Hong Kong SAR, China; Email: haoxiaoqiang168@yahoo.com.cn

**Keywords:** *Gekko swinhonis Guenther*, sulfated glycopeptide, hepatocellular carcinoma, migration

## Abstract

*Gekko swinhonis Guenther* has been used as an anti-cancer drug in traditional Chinese medicine for hundreds of years. Previous studies showed that the *Gekko* sulfated polysaccharide-protein complex suppressed the proliferation and migration of hepatoma cells. *Gekko* sulfated glycopeptide α was obtained from *Gekko* sulfated polysaccharide-protein complex using papain hydrolysis. *Gekko* sulfated glycopeptide α inhibited the proliferation and migration of SMMC-7721 cells. The secretion of IL-8 and the concentration of intracellular calcium were decreased after *Gekko* sulfated glycopeptide α exposure. SMMC-7721 cells in the control group showed abnormal features, with a polygonal shape, whereas this changed to a spindle shape after the treatment with *Gekko* sulfated glycopeptide α. Actin ﬁlaments were distributed diffusely along the cell membrane in control cells, whereas those were polymerized and preferentially accumulated in the cytoplasm of treated cells. Microtubules distributed in the cytoplasm of untreated cells were located diffusely whereas those in treated cells were polymerized. Therefore, *Gekko* sulfated glycopeptide α inhibit the migration of hepatoma cells via reducing the secretion of IL-8 and the concentration of intracellular calcium, as well as regulating the reorganization of cytoskeleton.

## 1. Introduction

A malignant tumor is a disease in which many characteristics of normal cell biological behavior are lost or destroyed. Excessive proliferation and inappropriate migration are the primary cause of death of cancer patients [1], so the development of oncotherapeutic drugs that prevent tumour proliferation and migration is urgently needed. Recent research has shown that sulfated polysaccharides and/or glycoproteins could inhibit the proliferation and metastasis of tumor cells [2,3,4,5]. Some glycopeptides obtained from glycoproteins by the digestion of trypsin and papain were demonstrated to have similar inhibitory effects on hepatocellular carcinoma (HCC) migration as the corresponding glycoproteins [6].

The whole body of *Gekko swinhonis* Guenther, commonly known as Gecko, has been used as an anti-cancer drug in traditional Chinese medicine for hundreds of years. The gecko is an animal of the genus Gekko, family of Gekkonidae, which is widely spread throughout the north and central regions of China. We extracted a sulfated polysaccharide-protein complex from *Gekko swinhonis* Guenther, which was named Gekko sulfated polysaccharide-protein complex (GSPP). Further research showed that it had strong effect on HCC cell migration via calcium-mediated regulation of the actin filaments reorganization, whereas no signiﬁcant effect on the secretion of interleukin-8 (IL-8). The trypan blue exclusion assay showed that GSPP at 200 µg/mL and 100 µg/mL can inhibit the proliferation of SMMC-7721 cells [7,8].

Sulfated polysaccharides can show improved anti-tumor activity [9], therefore we obtained a new sulfated glycopeptide named GSPP α using papain hydrolysis, which had lower molecular weight (MW) and more sulfate content than GSPP. This research focuses on whether GSPP α has better anti-cancer activity than GSPP after the structural modification and its underlying mechanism of action.

## 2. Results and Discussion

### 2.1. Homogeneity Analysis

**Figure 1:**
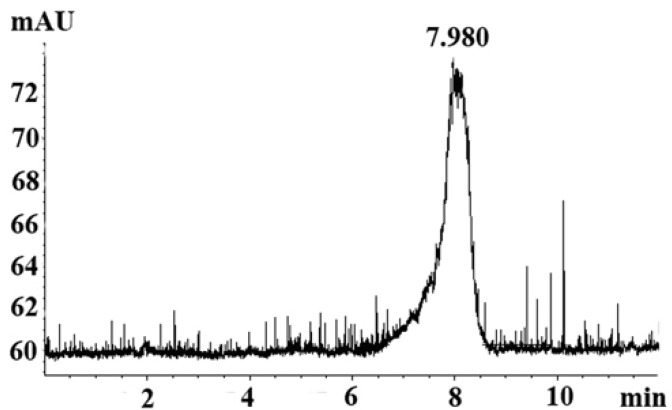
HPLC proﬁle for *Gekko* sulfated glycopeptides. There was a single and symmetrically sharp peak at 7.98 min in the HPLC proﬁle.

GSPP α was obtained from GSPP using papain hydrolysis. There was a single and symmetrically sharp peak at 7.98 min in high performance liquid chromatography (HPLC) proﬁle, which suggested that GSPP α was homogeneous (Figure 1). In the HPLC proﬁles, GSPP showed a peak at 5.88 min, which indicated that the MW of GSPP α was smaller than that of GSPP after the digestion with papain [7]. The MW of GSPP α was estimated to be over 2,000 kDa as judged from the calibration curve prepared from standard dextrans. The optical rotation [α]_D_^20^ of GSPP was −120° while the optical rotation [α]_D_^20^ of GSPP α was −60°.

### 2.2. Fourier Transform Infrared Spectrum (FTIR) Analysis

**Figure 2:**
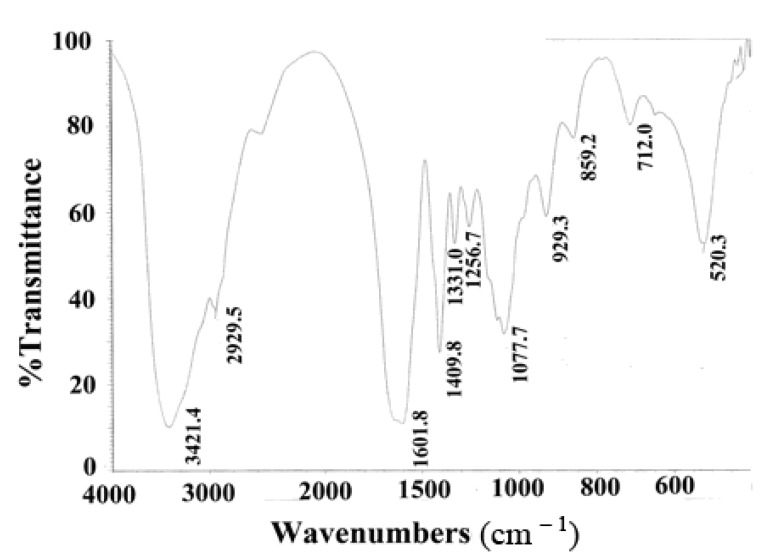
FTIR of *Gekko* sulfated glycopeptides. FTIR spectra showed that *Gekko* sulfated glycopeptides mainly contained four types of groups, namely hydroxyl group, methyl group, amido group and sulfate group.

The FTIR spectrum of GSPP α showed a major peak at 3,421.4 cm^−1^, weak bands at 2,929.5, 1,409.8, 1,256.7 and 859.2 cm^−1^, and a strong band at 1,601.8 cm^−1^. The peak at 3,421.4 cm^−1^ was characteristic of the O–H stretching vibration due to the hydroxyl group, while the bands at 2,929.5 and 1,409.8 cm^−^^1^ were characteristic of the stretching vibration and bending vibration of the C–H of the methyl group. The band at 1,601.8 cm^−1^ was due to the N–H bending vibration of the amido group, which indicated the presence of glucosamine. The bands at 1,256.7 and 859.2 cm^−1^ were ascribed to the stretching vibration of the S–O and C–O–S of sulfate group (Figure 2). Spectrophotometry showed that the sulfate content of GSPP α was 14.7%, whereas GSPP contained around 8.65% sulfate groups [7].

### 2.3. Amino Acid Composition and Glycopeptide Linkage Analysis

Amino acid analysis indicated that GSPP α contained 16 types of amino acids. A solution of the GSPP α treated with NaOH had a more dramatical absorption at 240 nm than the non-treated puriﬁed compound (data not shown). The content of threonine decreased from 7.12 to 3.64 µg per mg, while that of aminobutyric acid increased from 0 to 1.16 µg per mg. Thus, GSPP α?comprised *O*-glycopeptide linkages.

### 2.4. Effects of GSPP α on the Proliferation and Cell Cycle Distribution of SMMC-7721 Cells

**Figure 3:**
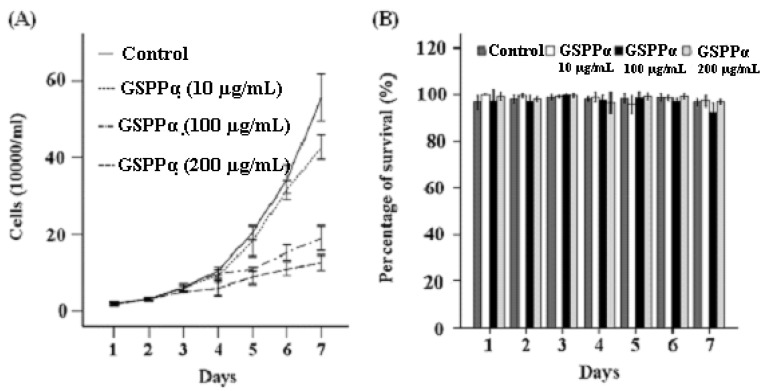
Effects of *Gekko* sulfated glycopeptides on proliferation and survival of SMMC-7721 cells. After exposure to *Gekko* sulfated glycopeptides, SMMC-7721 cells were collected at 30 min, then 24, 48, 72, 96, 120 and 144 h. The total cell and viable cell counts were determined by using a trypan blue exclusion assay. The growth curves of cells (**A**) and percentage of survival (**B**) were plotted. Raw data for the survival were expressed as the percentage of survival. The values represent means ± SD. GSPP α: *Gekko* sulfated glycopeptides.

Trypan blue exclusion assay and flow cytometry assay showed that GSPP α can more effectively inhibit the proliferation of SMMC-7721 cells and block cells in the S phase than GSPP. GSPP α at each concentration (10 μg/mL, 100 μg/mL and 200 μg/mL) was capable of inhibiting the proliferation of SMMC-7721 cells. Even at a concentration of 10 μg/mL, GSPP α inhibited the proliferation of SMMC-7721 cells strongly (*P* < 0.001, Figure 3A). However, GSPP only possessed an inhibitory effect at the concentrations of 100 μg/mL and 200 μg/mL. Ten μg/mL GSPP had no effect on the malignant proliferation of SMMC-7721 cells [7]. Besides, the percentage of S-phase cells in the group treated with 100 µg/mL and 200 µg/mL GSPP α was much higher than that of the control group (*P* = 0.001, *P* = 0.013, data not shown). However, GSPP α had no effect on the survival of SMMC-7721 cells (Figure 3B).

### 2.5. Effects of GSPP α on the Migration of SMMC-7721 Cells

**Figure 4:**
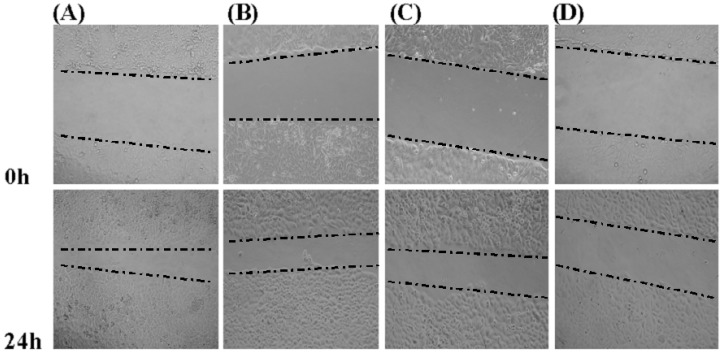
Wound-healing assay of *Gekko* sulfated glycopeptides on SMMC-7721 cells. Phase-contrast microscope was used to observe the linear wound of the cells (100×). Indicated times (0, 24 h) depict the duration after scratching of the monolayer. (**A**) Control group; (**B**) 10 µg/mL *Gekko* sulfated glycopeptides; (**C**) 100 µg/mL *Gekko* sulfated glycopeptides and (**D**) 200 µg/mL *Gekko* sulfated glycopeptides.

**Figure 5:**
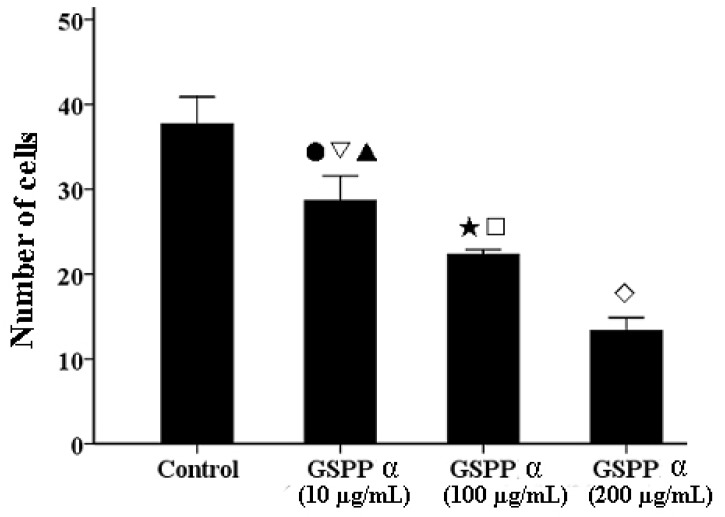
Transwell assay of *Gekko* sulfated glycopeptides on SMMC-7721 cells. After staining with crystal violet, cells that had penetrated through the chamber were counted using a light microscope. Each bar showed the number of cells that had penetrated throughthe chamber (*P* < 0.001). The values represent means ± SD. ● *versus* control group, *P* = 0.001; **▽** *versus* 100 µg/mL GSPP α, *P* = 0.01; ▲ *versus* 200 µg/mL GSPP α, *P* < 0.001; ★ *versus* control group, *P* < 0.001; **□** *versus* 200 µg/mL GSPP α, *P* = 0.001; **◇** *versus* control group, *P* < 0.001. GSPP α: *Gekko* sulfated glycopeptides.

We investigated the effect of GSPP α on cell migration via wound-healing assay. GSPP α down regulated SMMC-7721 cell migration in a dose-dependent manner, as compared to the control cells (Figure 4). To further conﬁrm the inhibitory effect of GSPP α on cell migration, a transwell assay was performed. GSPP α dramatically inhibited cell migration, compared with the untreated controls (*P* < 0.001). Even at 10 µg/mL, the inhibitory effect was obvious (Figure 5). Some glycopeptides obtained from glycoproteins by the digestion of trypsin and papain were demonstrated to have similar inhibitory effect on HCC migration as the corresponding glycoproteins [6]. Wound-healing assay and transwell assay indicated that GSPP α had a more signiﬁcant effect than GSPP on inhibiting the migration of SMMC-7721 cells.

### 2. 6. Effects of GSPP α on The IL-8 Secretion from SMMC-7721 Cells

**Figure 6:**
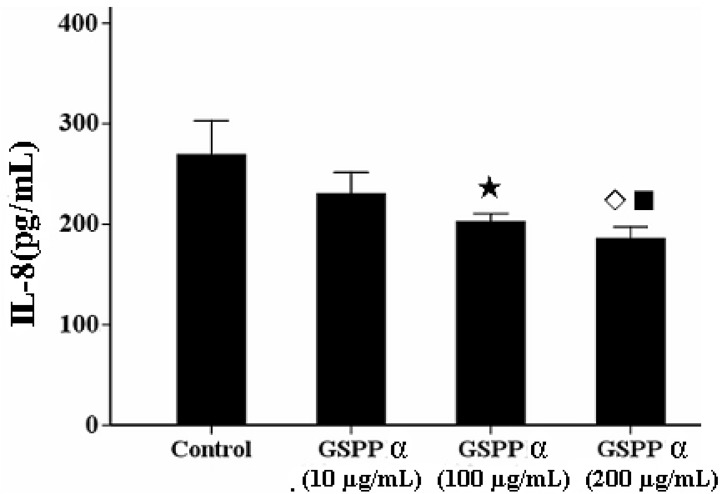
Effect of *Gekko* sulfated glycopeptides on IL-8 secretion. The values represent means ± SD. *P* = 0.006. ★ *versus* control group, *P* = 0.005; ◇ *versus* control group, *P* = 0.001; ■ *versus* 10 µg/mL GSPP α, *P* = 0.032. GSPP α: *Gekko* sulfated glycopeptide.

IL-8 has the capacity to lead to the tumor migration via regulating the concentration of intracellular calcium and the reorganization of cytoskeleton [6,10,11]. GSPP at any concentration was not effectively against the secretion of IL-8 from SMMC-7721 cells. However, GSPP α at the concentrations of 200 and 100 µg/mL are capable of downregulating the IL-8 secretion from SMMC-7721 cells compared with the control cells (*P* = 0.006) (Figure 6).

### 2.7. Effects of GSPP α on the Intracellular Calcium in SMMC-7721 Cells

**Figure 7:**
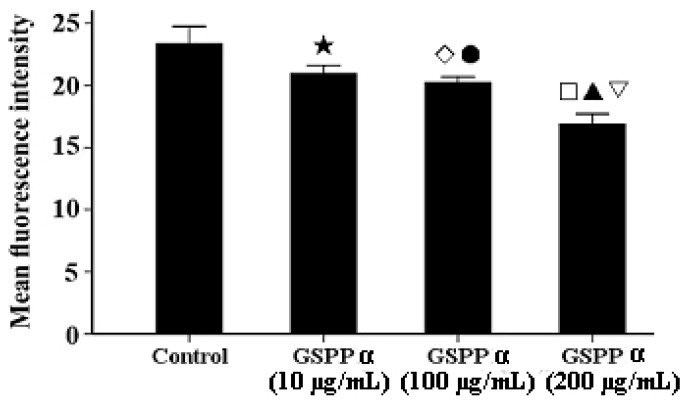
Effect of *Gekko* sulfated glycopeptides on the intracellular calcium concentration in SMMC-7721 cells. Cells were incubated with the Fluo-3 AMester and analyzed by ﬂow cytometry. The values represent means ± SD. ★ *versus* control group, *P* = 0.011; ◇ *versus* control group, *P* = 0.003; ● *versus* 200 µg/mL GSPP α, *P* = 0.002; **□** *versus* control group, *P* < 0.001; ▲ *versus* 10 µg/mL GSPP α, *P* = 0.001; ▽ *versus* 100 µg/mL GSPP α, *P* = 0.002. GSPP α: *Gekko* sulfated glycopeptides.

Many recent studies have shown that tumor cell migration is often downregulated by decreasing intracellular calcium concentrations, concomitant with cytoskeleton reorganization [12,13]. Calcium can regulate the polymerization and reorganization of cytoskeleton via regulating the function of cytoskeleton binding proteins [14]. As shown in Figure 7, treatment with GSPP α resulted in a signiﬁcant decrease of intracellular calcium content in SMMC-7721 cells in dose-dependent manner (*P* < 0.001).

### 2.8. Effects of GSPP α on the Cytoskeleton in SMMC-7721 Cells

The cytoskeleton provides the basic infrastructure for the maintenance of cell motility. It is known that there are three types of cytoskeletal proteins: Microtubules, intermediate ﬁlaments, and actin ﬁlaments [13]. Tumor cell migration depends mainly on the polymerization and reorganization of actin filaments and microtubules. The protrusive lamellipodias at the cell front are formed via actin polymerization, followed by the occurance of the retractable tail at the cell rear via microtubules reorganization [15]. Through generating both pushing and contractile forces, actin filaments and microtubules are capable of promoting cell motility [1].

**Figure 8:**
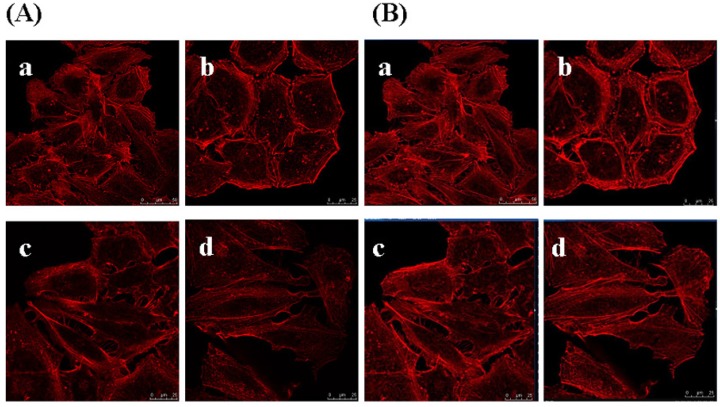
Effect of *Gekko* sulfated glycopeptides on actin filament. Cells were incubated with the phalloidin rhodamine and photographed under oil microscope (600×). (**A**) Sectional images (on the left); (**B**) 3-dimensional images (on the right). (**a**) Control group; (**b**) 10 µg/mL *Gekko* sulfated glycopeptides; (**c**) 100 µg/mL *Gekko* sulfated glycopeptides and (**d**) 200 µg/mL *Gekko* sulfated glycopeptides.

**Figure 9:**
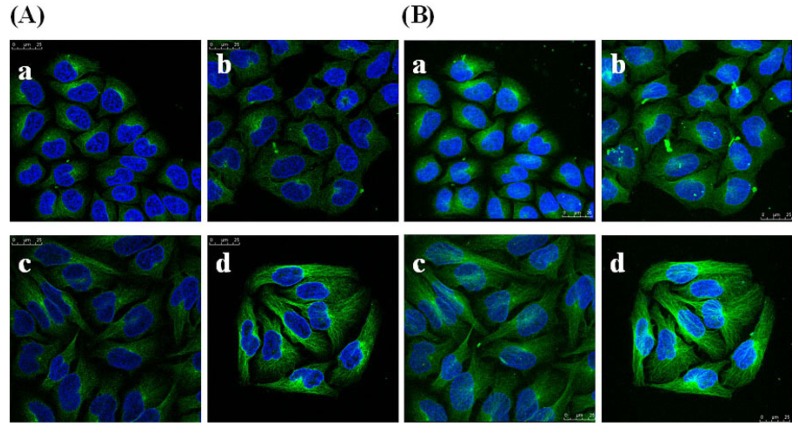
Effect of *Gekko* sulfated glycopeptides on the microtubules of SMMC-7721 cells. Cells were first incubated with antibody against microtubules and then fluorescence-conjugated secondary antibody against the first antibody, followed by staining with 4’,6-diamidino-2-phenylindole and photography. Then the cells were photographed under oil microscope (600×). (**A**) Sectional images (on the left); (**B**) 3-dimensional images (on the right). (**a**) Control group; (**b**) 10 µg/mL *Gekko* sulfated glycopeptides; (**c**) 100 µg/mL *Gekko* sulfated glycopeptides and (**d**) 200 µg/mL *Gekko* sulfated glycopeptides.

To determine whether GSPP α has an effect on the cytoskeleton in SMMC-7721 cells, we studied the localization and conﬁguration of actin ﬁlaments and microtubules. SMMC-7721 cells in control group showed abnormal feature with a polygonal shape. Modiﬁcations of cell shape were induced by GSPP α. SMMC-7721 cells changed to a spindle shape after the treatment with GSPP α. As shown in Figure 8, actin ﬁlaments in control cells were distributed diffusely along the cell membrane, whereas those in treated cells were polymerized and preferentially accumulated in the cytoplasm. Besides, microtubules in both untreated cells and treated cells were distributed in the cytoplasm. However, microtubules in untreated cells were diffused where as those in treated cells were polymerized (Figure 9).

## 3. Experimental

### 3.1. Cell Culture and Reagents

The cellulose dialysis bag (MWCO 3500Da) was purchased from Union Carbide (USA). Standard dextrans (T10, T40, T70, T110, T500 and T2000) were purchased from Amersham Pharmacia (Sweden). Human HCC cell line SMMC-7721 was obtained from the Shanghai Institute of Cell Resource Center, Chinese Academy of Life Science. It was certiﬁed by associate professor Xiong-Zhi Wu from Tianjin Medical University Cancer Institute and Hospital to ensure that they were free of contamination and the designated type. Cells were maintained in DMEM medium (Gibco, USA) supplemented with 10% fetal bovine serum (Sigma, USA), 50 U/mL penicillin and 50 mg/mL streptomycin. Then the cells were cultured with 95% humidity and 5% CO_2_ at 37 °C. SMMC-7721 cells were seeded in 12-well plates at 0.5 × 10^4^/mL. Then the plates were incubated for 24 h in a cell incubator before being exposed to GSPP α (10, 100 and 200 µg/mL, respectively). For the control group, an equal volume of DMEM was added into the medium. A IL-8 enzyme-linked immunosorbent assay kit was purchased from R&D systems (USA). Fluo-3 AM ester was purchased from Invitrogen (USA) and phalloidin rhodamine was purchased from Molecular Probes (Carlsbad, CA). Antibody for microtubules staining were purchased from Boster (China). Secondary antibody was fluorescein isothiocyanate anti mouse-IgG (Boster, China). CXCL12 was purchased from ProSpec-Tany TechnoGene Ltd (Israel). Transwell chambers were purchased from Corning (USA).

### 3.2. Preparation of GSPP α

GSPP was isolated from the dried whole body of *Gekko swinhonis* Guenther by water extraction and ethanol precipitation, followed by removal of free protein and dialysis [7]. Then the new compound GSPP α was obtained from GSPP using protease hydrolysis [6,16]. Brieﬂy, GSPP (10 g) was dissolved in phosphate buffer solution (1000 mL, pH 6.5, 0.1 mol/L), which contained 100 mg papain, 0.5 mol/L sodium chloride, 10 mmol/L-cysteine and 10 mmol/L-edathamil disodium. The reaction mixture was incubated in a water bath at 65 °C for 12 h, followed by the supplement with 50 mg papain at 65 °C for an additional 12 h. Then the reaction mixture was concentrated and ethanol precipitated, followed by the removal of free protein via the Sevage method [17]. Then the solution was dialyzed against distilled water for 24 h to remove those materials with molecular weight less than 3,500 Da. The material inside the dialysis bag was ﬁltered and dried to obtain GSPP α.

### 3.3. HPLC Analysis

The GSPP α solution was injected on an Agilent 1,100 series apparatus, equipped with a Shodex KS-805 column (Shoko, Japan), eluted at 1 mL/min flow rate with distilled water at 35 °C. To test the MW of GSPP α, T-series dextrans were passed successively through the calibrated column, and their retention times were plotted according to the logarithm of their respective MWs. The MW of the GSPP α was estimated via plotting its retention time in the same graph [18].

### 3.4. General Analysis of GSPP α

Uronic acid content was determined according to the Blumenkrantz and Asboe-Hansen method, using galacturonic acid as the standard [19]. Sulfate group content was measured by spectrophotometry as described by Dodgson and Price [20]. Besides, amino acid composition was determined using an amino acid analyzer (HITACHI 835, Japan). The optical rotation of GSPP α was determined on a Perkin-Elmer 243B polarimeter. The infrared spectrum was recorded with dried sample using a FTIR spectrophotometer (NEXUS-470, USA).

### 3.5. β-Elimination Reaction

GSPP α was dissolved in a solution containing 0.3 mol/L NaOH, 1 mol/L NaBH_4_. The solution was incubated at 45 °C for 24 h, followed by supplementation with 25% acetic acid. Both solutions before and after the treatment with NaOH were scanned from 200 nm to 400 nm by UV-Vis spectrophotometer (CARY 300 BIO, Varian) and then analyzed using the amino acid analyzer.

### 3.6. Trypan Blue Exclusion Assay and Flow Cytometry Analysis

After exposure to GSPP α, SMMC-7721 cells were collected at 30 min, then 24, 48, 72, 96, 120 and 144 h. The total cell and viable cell counts were determined using a trypan blue exclusion assay. Then, the growth curve of SMMC-7721 cells was drawn. The percentage of cell survival was calculated as follows: percentage of survival (%) = (mean viable cell number / mean total cell number) × 100%. To conﬁrm the further effect of GSPP α on cell cycle distribution, cells were stained with propidium iodide after treatment with GSPP α for 5 days. Then cell DNA was analyzed by ﬂow cytometry (BD Biosciences, USA). To analyze intracellular calcium concentration, cells were incubated with the Fluo-3 AM ester (5 µmol/L) for 40 min at 37 °C. The intracellular calcium content was then measured using ﬂow cytometry and expressed as mean ﬂuorescence intensity.

### 3.7. Wound-Healing Assay and Transwell Assay

A pipette tip about 1 mm in width was used to scratch the monolayer cell culture after cell conﬂuency. Then the DMEM medium containing GSPP α (0, 10, 100 and 200 µg/mL) and 10% FBS were added for cell growth. Phase-contrast microscope was used to observe the linear wound of the cells after 24 h. Migration assay using a Millipore chamber was performed as described by Yang [21]. Four days after exposure to GSPP α, cells were serum-starved for 24 h, trypsinized, and resuspended in serum-free DMEM medium. Around 1 × 10^5^ cells were seeded into the upper chamber, with the lower chamber supplemented with the DMEM medium containing 10% FBS and 100 ng/mL CXCL12. Then the chamber was incubated for 48 h at 37 °C to allow the possible migration of cells. Membranes were stained with crystal violet according to manufacturer’s recommendation. Then the cells on the upper chamber were removed. The cells that had penetrated through the membrane were counted using a light microscope.

### 3.8. ELISA

The secretion of IL-8 in cell supernatant was measured by using the enzyme-linked immunosorbent assay kit according to the manufacturer’s protocol. IL-8 concentration was estimated via extrapolation from the IL-8 standard curve.

### 3.9. Confocal Microscopy

Five days after the treatment with GSPP α, cells cultured on coverslips were ﬁxed with 4% paraformaldehyde, followed by permeabilization with 0.1% Triton X-100. The cells were then labeled with phalloidin rhodamine in blocking buffer at 4 °C overnight. Besides, to determine the conﬁguration of microtubules, cells were incubated with first antibody against microtubules at 4 °C overnight, and fluorescence-conjugated secondary antibody against first antibody at room temperature for 1 h, followed by staining with 4’,6-diamidino-2-phenylindole [22]. Immunostained cells were then examined using a confocal microscope (Leica TCS SP5 MP, Germany).

### 3.10. Statistics and Data Analysis

All experiments were repeated three times. Single-factor ANOVA and general linear model were performed to analyze data. Statistical calculations were performed by SPSS (Version: 13.0, Chicago, USA). *P* < 0.05 was considered statistically significant. All values were expressed as means ± SD.

## 4. Conclusions

In summary, a new compound was obtained from Gekko sulfated glycoprotein by the digestion with papain and chemically characterized as Gekko sulfated glycopeptide α (GSPP α). GSPP α had less MW and more sulfate content than GSPP. Further research demonstrated that GSPP α had more significant inhibitory effects on the proliferation and migration of SMMC-7721 cells than GSPP. Moreover, GSPP α could inhibit the migration of SMMC-7721 cells in a dose dependent manner by reducing the secretion of IL-8 and the concentration of intracellular calcium, as well as regulating the reorganization of cytoskeleton.
